# Transoral Endoscopic-Assisted Adenoidectomy: A Prospective Study

**DOI:** 10.1055/s-0045-1811954

**Published:** 2026-03-03

**Authors:** Mohamed Zahran, Ahmed Ashour

**Affiliations:** 1 Department of Otolaryngology-Head and Neck Surgery, Alexandria University Hospital, Alexandria, Egypt; 2 Department of Ear, Nose, and Throat, Zain Hospital, Kuwait

**Keywords:** adenoid, adenoidectomy, curettage adenoidectomy, endoscope, nasopharynx, adenoid recurrence

## Abstract

**Introduction:**

Adenoidectomy, with or without tonsillectomy, is a common pediatric surgical procedure. It has been noted that blind curettage has many drawbacks. First of all, it cannot completely remove the adenoid tissue from the posterior choana or the margins of the Eustachian tube orifices, which leads to incomplete removal. Secondly, applying curettage blindly may damage the Eustachian tube opening.

**Objective:**

To illustrate our surgical technique for a successful and safe adenoidectomy using a transoral 70° endoscope.

**Methods:**

The present was a prospective study carried out from December 2022 to September 2024. All patients had adenoid hypertrophy either alone or with tonsillar hypertrophy. There were 46 female and 50 male patients, all of whom underwent transoral endoscopic adenoidectomy using a 70° scope.

**Results:**

There were 96 patients included in this study. The mean age was 5 years and 8 months. Of that total, 35 cases were revisions. The primary procedure was done 12 to 26 months prior (mean: 19). All patients were compliant for the scheduled fiberoptic scope 1 year after surgery. We found that 4 patients (4.16%) had small adenoid regrowth, primarily near the Eustachian tube orifices, without causing any symptoms or airway compromise.

**Conclusion:**

Transoral endoscopic adenoidectomy is a technically viable procedure that is better than the traditional curettage method. There was reduced likelihood of incomplete adenoid removal, and it is nearly impossible to damage adjacent structures, making it safer than blind curettage.

## Introduction

The adenoid is a component of the Waldeyer's ring of lymphoid tissue located at the entrance of the upper respiratory tract. Anatomically, adenoid tissue is found in the nasopharyngeal posterior wall's roof. The nasopharyngeal lymphoid aggregation, also known as Luschka's tonsil, was first described by Santorini in 1724. In 1870, Wilhelm Meyer first used the term “adenoid” to describe “nasopharyngeal vegetations.” 1

In younger children, it seems to play a significant role in the establishment of “immunological memory”. Early childhood naturally acquired immunity is significantly influenced by oral and nasal antigen exposure. There are B-cells produced by adenoid, which develop into IgG and IgA plasma cells. 2

Adenoid hypertrophy is a prevalent issue in this population, causing recurrent ear effusions, chronic rhinorrhea, snoring, and obstructive sleep apnea. Poor academic achievement and behavioral problems are associated with the latter. 3 4

Adenoidectomy, with or without tonsillectomy, is a common pediatric surgical procedure. The clear indication for the surgery is adenoid enlargement that results in snoring, sleep apnea, and persistent secretory otitis media. 5 Usually, the procedure reduces the symptoms, but some patients may experience recurrence. Numerous risk factors, including young age, allergic rhinitis, reflux, and inadequate tissue excision, have been linked to adenoid regrowth. 6

Researchers are still looking for ways to improve adenoidectomy performance and postoperative recovery. Ease of application, including efficiency and intraoperative hemostasis are characteristics of an ideal approach. Of course, the question of which technique produces the best results is still up for debate. 7

Novel techniques for adenoidectomy have been proposed, including the use of microdebriders, electrocautery, and nasal endoscopy. 8 9 The most common adenoidectomy surgical technique is still the conventional one with curettage.

It has been noted that blind curettage has many drawbacks. First, it cannot completely remove the adenoid tissue from the posterior choana or the margins of the Eustachian tube orifices, which leads to an incomplete procedure. Second, applying curettage blindly carries the danger of damaging the Eustachian tube opening. Third, improper visualization of the bleeding spots leads to increased postoperative bleeding and the placement of adenoid packs. 10

The aim of the present article is to illustrate the feasibility and efficacy of adenoidectomy using transoral 70° endoscope and curved suction electrical coagulator.

## Methods

The present was a prospective study carried out in the period from December 2022 to September 2024 including 96 patients. All of them had adenoid hypertrophy either alone or with tonsillar hypertrophy. There were 46 female and 50 male patients. The mean age was 5 years and 8 months, with a range of 2 years and 8 months to 9 years. Of the total, 35 cases were revision cases. The primary procedure was done between 12 and 26 months (mean: 14).

The present study was approved by hospital ethics committee (2024/2024-108), and informed consent was obtained from patients' parents, adhering to the standards established by the Declaration of Helsinki.

All patients were subjected to history analysis, which included symptoms related to adenoid hypertrophy, disease course and duration, aggravating or relieving factors, association with recurrent tonsillitis or hearing problems. Furthermore, history of allergic rhinitis and bronchial asthma, medication use or allergies, and previous surgeries (adenoidectomy, tonsillectomy, cleft palate repair etc.) were considered.

Physical examination consisted of the standard ear, nose, and throat (ENT) inspection with otoscopy and fiberoptic nasopharyngoscopy to directly visualize the adenoid and exclude any associated nasal/nasopharyngeal abnormalities.

We investigated the plain X-ray nasopharynx (lateral film), which was reserved for uncooperative children in whom scope could not be performed, as well as tympanometry if secretory otitis media was suspected, and routine blood investigations (complete blood count [CBC], coagulation etc.).

### Exclusion Criteria

Patients with blood or coagulation disorders, cleft palate, those unfit for general anesthesia, and those who missed postoperative follow-ups were excluded from the study.

### Operative Details

**Fig. 1:**
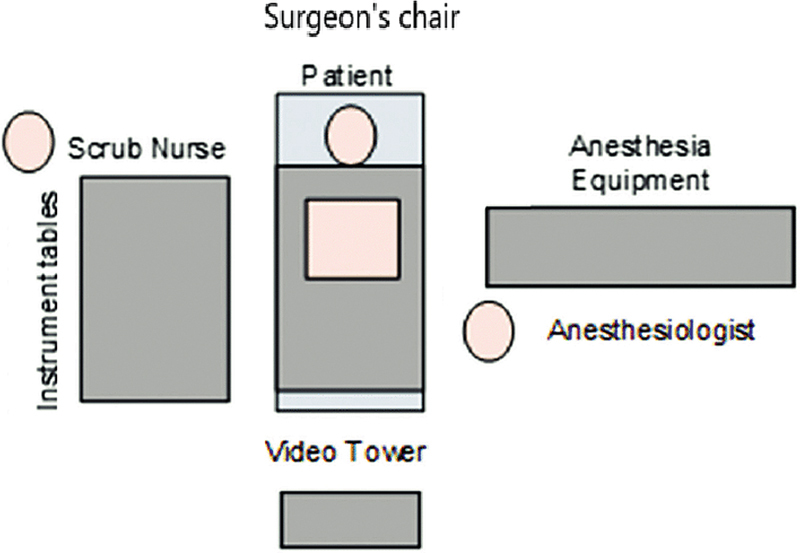
Operating room (OR) setup.

The operating room (OR) setup is demonstrated in Fig. 1 . All patients underwent general anesthesia using oral endotracheal intubation, adjusted by size according to patients' ages. A Boyle-Davis mouth gag was inserted gently to open the mouth after lubricating the lips with saline or moisturizing jelly to prevent any mucosal breaks in dry lips.

To retract the soft palate, artery forceps were used to hold the ends of two rubber catheters that had been inserted through each nostril and into the mouth.

The adenoid mass is visualized after the 70° Hopkins 4-mm nasal endoscope is inserted through the mouth. The endoscopic image is displayed on a monitor, as a camera is mounted on the endoscope (Karl Storz GmbH & Co KG).

**Fig. 2:**
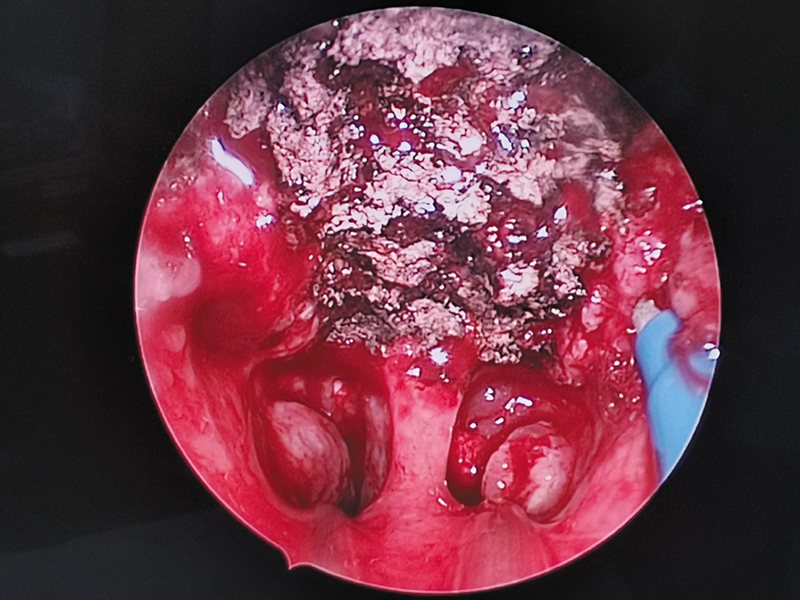
The field of the nasopharynx after adenoid removal by suction cautery.

**Fig. 3:**
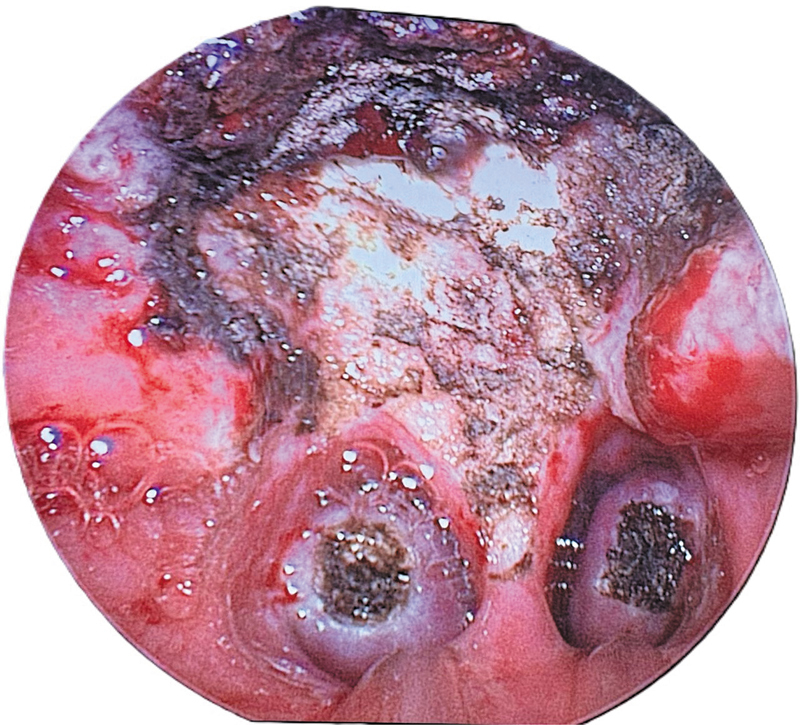


The adenoidectomy was done under vision using a Valleylab transoral curved suction electrical coagulator and energy platform (Medtronic PLC.). The field of the nasopharynx after adenoid removal by suction cautery is shown in Figs. 2 , 3 .

In patients with tonsillar hypertrophy or chronic tonsillitis, tonsillectomy was then performed using the cold steel technique and bipolar cautery for hemostasis (n = 48). Myringotomy with ventilation tube insertion was done in 52 patients.

### Postoperative Instructions

Routine postoperative medications (analgesics, antibiotics) were prescribed. Parents were instructed to return to the ENT causality department in case of persistent nasal and/or oral bleeding. A follow-up visit was scheduled between 7 and 10 days after the procedure.

Parents were directed to come back if their children experienced return of nasal obstructive symptoms other than the classic flu. Otherwise, they were asked to come for follow-up 1 year after surgery. At this follow-up, fiberoptic nasopharyngoscopy was done to check any recurrent adenoid.

## Results

A total of 96 patients were included in this study, 46 of whom were female and 50 males. The mean age was 5 years and 8 months, with a range of 2 years and 8 months to 9 years. All patients had adenoid hypertrophy, either alone or with tonsillar type (n = 48). Myringotomy with ventilation tube was done in 31 patients. The postoperative period was uneventful for all patients, with no reports of postoperative bleeding or other complications.

There were 35 revision cases. The primary procedure was done 12 to 26 months (mean: 19) before the study. According to patients' surgical records, the primary adenoidectomy was done by curettage either blindly (n = 15) or under indirect mirror view (n = 20).

All patients were compliant for the scheduled fiberoptic scope 1-year after surgery. Furthermore, 4 patients (4.16%) had small adenoid regrowth, primarily near the Eustachian tube orifices, without causing any symptoms or airway compromise. No further treatment was needed.

## Discussion

Meyer made the first reference to adenoidectomy in 1867. 11 By palpating the “nasopharyngeal vegetations,” he blindly placed a sharp ring curette into the oral cavity. In 1885, Gottstein designed the first ring knife. 12 In 1897, Beckmann made modifications to this ring knife. 13 To remove adenoid bulk, punch forceps and an adenotome were also developed. The renowned adenoidectomy curette, with a cage to guarantee safety and entangle the blood and tissue pieces during the procedure, was created by Sir St. Clair Thomson. The traditional curettage adenoidectomy still employs this tool. 14

Curettage is currently a standard technique for blind adenoidectomy. The St. Clair Thompson tool is engaged at the adenoid pad after being inserted into the oral cavity, avoiding the oropharynx to reach the nasopharynx. One pass of the curette removes the adenoid mass, from superior to inferior. Injury to the torus region, choanal area, or deep muscle should be avoided. To establish hemostasis, a pack is put into the resulting adenoid bed. Digital palpation is used to verify completion. 15

Adenoidal remnants or regrowth are very common findings after the procedure. Havas and Lowinger used intraoperative nasal endoscopy for proof that up to 39% of patients do not have adequate removal by curette adenoidectomy. 16 In 2004, Bross-Soriano et al. 17 assessed the traditional technique's effectiveness in removing all adenoidal tissue and found that it was less than 30%. There were 35 revision cases in the current study. Traditional curettage was the initial operation in each of the cases. The amount of residual and/or recurrent adenoid tissue was significant enough in each case to result in symptom persistence or recurrence.

Nofal et al. 18 conducted a prospective study with 100 children to evaluate the effectiveness of traditional curettage adenoidectomy. After the procedure, the nasopharynx was endoscopically examined using a 0° telescope to assess remnants, bleeding points, and injuries to the surgical field or nearby structures. Adenoid remnants were found in 42% of patients, including the posterior pharyngeal wall (4%), posterior end of the nasal septum (2%), tubal tonsil (12%), and roof of the nasopharynx over the choana (24%). In 46% of the instances, there was damage to the surgical field and surrounding structures (lateral or posterior pharyngeal wall and Eustachian tube orifice). Bleeding points were seen in 29% of cases, with adenoid remnants accounting for 13%, mucosa for 10%, and pharyngeal muscles for 6%.

A related prospective study comprised 1,900 children with adenoid enlargement who underwent transoral endoscopic evaluation after completing traditional curettage adenoidectomy. A 70° Hopkins 4-mm nasal endoscope was used to examine the adenoid bed through the mouth. Adenoid tissue remnants were seen and treated in 855 patients (45%) and bleeding sites in 17 cases (0.9%). 19

To assess the benefits of adjuvant transnasal endoscopic adenoidectomy following traditional curettage, another study was conducted. The authors used the 30° 4 mm rigid endoscope through the nose on the broader side. The 2.7 mm endoscope was used in narrow nasal passages. Adenoid remnants were detected in 92.15% of their patients following traditional curettage. The other nostril was used to introduce a shaver to remove adenoid remnants, followed by cauterization of any bleeding points. In contrast to our technique, the authors found that performing an entirely transnasal and endoscopical adenoidectomy with the shaver was more time-consuming and resulted in more bleeding. 20

To prevent any potential early damage from curettage, we performed the adenoidectomy entirely by transoral endoscopy. Under direct eyesight, all of the tissue was removed without the danger of leaving any residual behind. This took an additional 3 to 4 minutes, not considered time-consuming. The endoscope, camera, and monitor were all already functional in the operating room.

In a similar study, 300 children had transoral endoscopic adenoidectomy utilizing a 70∘ Hopkins 4-mm nasal endoscope, the traditional adenoid curette, and St. Claire Thomson's forceps. There were no reports of complications following the procedure. Children with recurrent obstructive nasal symptoms underwent flexible nasopharyngoscopy and were monitored via telephone questionnaire. Due to adenoid regrowth, only one patient experienced recurring obstructive nasal symptoms; further evaluation revealed this patient had a nasal allergy, which could be the reason for the recurrence. Likewise, no postoperative bleeding or other complications were reported during our study. After 1 year, four patients (4.16%) experienced minor adenoid regrowth, mostly in the vicinity of the Eustachian tube orifices, without any symptoms or airway compromise. No further treatment was required. 21

Using databases like PubMed/Medline, EMBASE, EBSCO, and the Cochrane Library, a systematic review of published papers was conducted in 2021. 22 We included all randomized controlled trials published in English between 1965 and 2021 that compared traditional curettage adenoidectomy with other surgical methods. In comparison to this technique, endoscopic-assisted microdebrider adenoidectomy produced a significantly higher estimate of intraoperative blood loss. Since suction diathermy was thought to generate the least amount of intraoperative blood loss, it had the highest cumulative likelihood of being chosen. However, the authors concluded that no single approach could be ideal for every possible outcome.

Our study's objective was to show that the transoral endoscopic adenoidectomy is more effective and superior to the traditional curettage method. We didn't compare various instruments, such as a microdebrider with a laser or suction cautery. Since suction cautery produced the best hemostasis and the least amount of intraoperative blood loss, it was used in our approach.

## Conclusions

Transoral endoscopic adenoidectomy is a technically viable procedure that is better than the traditional curettage method. There was reduced likelihood of incomplete adenoid removal. It is nearly impossible to damage adjacent structures, making it safer than blind curettage.
